# Beyond recovery: long-term cardiovascular risks after severe COVID-19 requiring intensive care

**DOI:** 10.1186/s13054-026-06088-5

**Published:** 2026-05-25

**Authors:** Johanna Kämpe, Martin Jonson, Rebecka Rubenson Wahlin, Jacob Hollenberg, Per Svensson, Per Nordberg

**Affiliations:** 1 Department of Clinical Science and Education, Centre for Resuscitation Science, Karolinska Institutet, Södersjukhuset, Stockholm, 17177 Sweden; 2 Department of Clinical Science and Education, Karolinska Institutet, Södersjukhuset, Stockholm, 17177 Sweden; 3 Department of Clinical Science, Intervention and Technology, Division of Anaesthesia and Intensive care, Karolinska University Hospital, Stockholm, 17177 Sweden; 4 Department of Physiology and Pharmacology, Karolinska Institute, Stockholm, Sweden

**Keywords:** Covid-19, Intensive care unit, Atherosclerotic cardiovascular disease, Cardiovascular outcomes, Propensity score matching, Long-term follow-up

## Abstract

**Background:**

Severe Covid-19 has been associated with acute cardiovascular complications, but data on long-term cardiovascular outcomes after critical care are limited. This study aimed to evaluate the risk of atherosclerotic cardiovascular disease (ASCVD) within three years following severe COVID-19 requiring intensive care.

**Methods:**

This nationwide, population-based matched cohort study included adults with confirmed COVID-19 who required mechanical ventilation in the intensive care unit (ICU) and were discharged alive from the hospital between March 1, 2020, and June 8, 2021, identified from the Swedish Intensive Care Registry. After a 1:4 propensity score match including age, sex, district of residence, comorbidities and socioeconomic factors - cases and controls were compared regarding cardiovascular outcomes during a three-year follow-up. The primary outcome was ASCVD events occurring more than 30 days after discharge from the ICU and secondary outcomes included hospitalization for heart failure, atrial fibrillation, and all-cause mortality.

**Results:**

After propensity score matching, 16,530 individuals (3,350 cases and 13,180 controls) were included. The median age was 61 years, and 71% were male. Compared with controls, cases had an increased risk for ASCVD [subdistribution hazard ratio (sHR) 1.42 (95% CI 1.26–1.60)], hospitalization with atrial fibrillation [sHR 1.85 (95% CI 1.64–2.10)] and heart failure [sHR 1.81 (95% CI 1.57–2.09)]. All-cause mortality [hazard ratio (HR) 1.48 (95% CI 1.26–1.74)] was also significantly more frequent among cases.

**Conclusion:**

Among ICU-treated survivors of severe COVID-19, the risk of ASCVD, heart failure, atrial fibrillation, and all-cause mortality was increased during three years of follow-up compared with the matched population-based controls, with associations strongest during the first year of follow-up [period-specific estimates are provided in the Supplementary].

**Supplementary Information:**

The online version contains supplementary material available at 10.1186/s13054-026-06088-5.

## Introduction

As of mid-2025, COVID-19 has caused more than 700 million confirmed cases and approximately 7 million deaths worldwide, according to the World Health Organization [1]. The long-term impact on global health, extending beyond the intense initial years of the pandemic, remains difficult to fully comprehend [2]. Observational studies have indicated that COVID-19 can lead to a range of cardiovascular diseases, including myocardial infarction, arrhythmias, heart failure and ischemic stroke [3]. Follow-up data suggest that COVID-19 may have implications in cardiovascular health, not only in the acute phase [4–7] but potentially persisting, at least one year after severe COVID-19 [8, 9]. However, the long-term trajectory of these conditions and the risk of new cardiovascular events remain less explored.

COVID-19 and cardiovascular disease share several established risk factors, including high age, male sex, obesity, diabetes, hypertension, and chronic kidney disease [10]. These shared risk factors may partly explain the observed associations between COVID-19 and cardiovascular outcomes. At the same time, severe infection may act as a trigger of underlying cardiovascular disease, complicating the distinction between pre-existing risk and infection-related effects.

Previous studies have shown that certain respiratory infections are associated with increased risk of acute myocardial infarction, stroke, and exaggerated heart failure, with influenza being the most studied pathogen [11–14]. Although the underlying mechanisms are not fully understood, proposed pathways are believed to involve pathogen specific mechanisms, and systemic inflammation [14, 15]. These mechanisms may be particularly relevant in critically ill patients in the intensive care unit, as they may be at increased risk. The long-term relationship between respiratory infections and cardiovascular disease also remains poorly characterized, particularly over follow-up periods extending several years.

In this nationwide observational study using high quality data from national health registries, the primary aim was to assess the risk for future atherosclerotic cardiovascular disease (ASCVD) in COVID-19 patients requiring mechanical ventilation at the ICU during a three-year follow-up. The secondary aim was to assess the risk for hospitalization with heart failure, atrial fibrillation, and all-cause mortality.

## Methods

Over a three-year follow-up period, the long-term cardiovascular consequences of severe COVID-19 were assessed in a nationwide study population of ICU-treated patients compared with population-based matched control subjects.

### Study population

Patients aged ≥ 18 years with severe COVID-19 who received mechanical ventilation and were discharged alive between March 1, 2020, and June 8, 2021 were identified from the Swedish Intensive Care Registry [16]. For each patient (case), ten population controls were randomly selected from the Swedish Population Register [17] and matched based on age, sex, and district of residence. The study database was linked to multiple mandatory national registries maintained by Statistics Sweden [17] and the National Board of Health and Welfare [18], using every individual’s unique personal identification number. Data was first collected at admission and again three years after the primary admission. Diagnoses, both primary and secondary, were collected from the National Patient Register within 15 years, and prescribed drugs within the 12 months, preceding ICU admission. If diagnoses emerged earlier than 30 days before ICU admission, they were defined as pre-existing and included as baseline covariates. [Details in Supplementary Figure S1-2]. Thereafter, the randomly selected controls were used to perform a propensity score match where we also included comorbidities (see below). All data was pseudonymized prior to analysis to protect personal integrity, and ethical approval was obtained from the Swedish Ethical Review Authority (Dnr 2020 − 01598).

### Study design

#### Exposure

The exposure was admission to an intensive care unit for COVID-19 and requiring mechanical ventilation. All cases had an ICU admission while none of the controls had been treated for COVID-19 in any Swedish intensive care unit.

#### Observation time

For cases, the index date was defined as ICU discharge followed by a 30-day period where no events were included. This was made to minimize the risk of including events related to the acute phase of COVID-19. For each matched control, the same index date was assigned based on the ICU-discharge date of the corresponding case. The 30-day period was applied identically to controls. Follow-up for both cases and controls began after this 30-day period, ensuring a same observation time and comparable risk periods across groups.

#### Outcome

Primary outcome was defined as ASCVD events during the three-year follow up period after being treated for severe COVID-19. ASCVD was defined as clinical manifestations of atherosclerosis. In line with European Society of Cardiology (ESC) guidelines [19], this includes ischemic heart disease and ischemic stroke. Similarly, the American Heart Association (AHA) [20] defines ASCVD as clinical coronary heart disease, including myocardial infarction and stable or unstable angina, as well as ischemic stroke. Secondary outcomes were hospitalization with heart failure (HF) and episodes of atrial fibrillation or flutter (AF). Ischemic heart disease (IHD) – defined as myocardial infarction and/or instable angina, and ischemic stroke were also analysed separately. We also used data from Swedish Intensive care Registry (SIR) [16] and the Swedish Cause of Death Registry [21] to determine all-cause mortality. All new events occurring more than 30 days after ICU discharge (see above) were included as outcomes. Since prior cardiovascular disease was not an exclusion criterion, cases may have experienced the same event previously, meaning outcomes reflect both incident and recurrent disease rather than first events only. Pre-existing conditions and events were accounted for as co-variates in the propensity score model.

### Propensity score matching

To reduce potential confounding and ensure a more balanced comparison between cases and controls, propensity score matching was performed based on relevant demographic and clinical covariates. In an attempted 1:4 match cases were matched with controls based on age, sex, civil status, region of birth, educational level, average yearly income, and the following pre-existing medical conditions: diabetes type I and II, obesity, chronic obstructive pulmonary disease (COPD), asthma, hypertension, atrial fibrillation, heart failure, ischemic stroke, ischemic heart disease, and chronic kidney disease. The propensity score was estimated using a multivariable logistic regression model including all listed covariates, without interaction terms. Patients with severe COVID-19 were matched to controls, using nearest-neighbour matching without replacement with a caliper width of 0.2 of the standard deviation of the logit of the propensity score. Individuals with missing data were excluded prior to matching. Covariate balance was assessed using standardized mean differences before and after matching, with values less than 0.1 indicating acceptable balance. Common support was confirmed by visual inspection of propensity score distributions before and after matching [Supplementary Figure S3]. Due to insufficient overlap in propensity scores not all cases could be matched to exactly four controls, the final matched population consisted of 3,350 cases and 13,180 controls, corresponding to 3.93 controls per case.

### Statistical methods

All statistical analyses were conducted using R version 4.4.1. Categorical variables are presented as frequencies and percentages, and continuous variables are reported as medians with interquartile range.

For non-fatal outcomes, competing risk analyses accounting for death as a competing event were performed using Fine-Gray subdistribution hazard models, robust sandwich variance estimators clustered by matched set were used to account for the correlation between individuals, and results are presented as subdistribution hazard ratios (sHR). Time-to-event analyses were assessed using Cox proportional hazards models stratified by matched set to account for the matched study design and presented as Hazard ratios (HRs) with 95% confidence interval (CIs). Cumulative incidence functions were used to estimate absolute risks over time.

The proportional hazards assumption was assessed for Cox models (all-cause death) using Schoenfeld residuals. For Fine-Gray subdistribution models, proportionality was evaluated visually using log-log plots of the cumulative incidence functions. As non-proportionality was identified for all outcomes, the overall sHR represent time-averaged estimates. Period-specific analyses at 0–1 year and 1–3 years are presented as the primary estimates to account for the time-varying nature of the associations [Supplementary].

Event rates were calculated per 1000 person-years. A two-sided p-value of < 0.05 was considered statistically significant.

## Results

In total, 4,921 patients with severe COVID-19 were identified, each initially matched with 10 population controls. After 1:4 propensity score matching, 3,350 patients and 13,180 controls were included in the final analysis [see flow chart in Supplementary]. Median age was 61 years old, and 71% were men. The prevalence of major cardiovascular risk factors – including hypertension (30%), type 2 diabetes (16%), chronic kidney disease (3.7%), and obesity (11%) – were similar between cases and controls after matching. Socioeconomic factors such as civil status, region of birth, educational level, and yearly income were after matching comparable across groups [Table 1 and Supplementary figure S3].

**Table 1:** Baseline characteristics of patients with severe COVID-19 (cases) and control characteristics after propensity score matching

Characteristic	Control (*n* = 13,180)^1^	Case (*n* = 3,350)^1^
**Age (years)**	61 (50, 70)	61 (52, 69)
**Sex (Male)**	9,311 (71%)	2,340 (71%)
**Medical conditions**		
Diabetes type 2	2,049 (16%)	541 (16%)
Diabetes type 1	414 (3.1%)	115 (3.4%)
Hypertension	3,879 (29%)	1,021 (30%)
Obesity	1,209 (9.2%)	357 (11%)
Asthma	887 (6.7%)	241 (7.2%)
Heart failure	592 (4.5%)	158 (4.7%)
Atrial fibrillation	827 (6.3%)	212 (6.3%)
Ischemic heart disease	619 (4.7%)	147 (4.4%)
Chronic Kidney Disease	427 (3.2%)	123 (3.7%)
Ischemic stroke	490 (3.7%)	97 (2.9%)
COPD	400 (3.0%)	106 (3.2%)
**Income (SEK)**	237,300 (161,200, 325,000)	232,300 (156,700, 322,800)
**Civil status**		
Divorced	2,382 (18%)	622 (19%)
Married	7,274 (55%)	1,845 (55%)
Unmarried	3,011 (23%)	755 (23%)
**Region of birth**		
Sweden	8,041 (61%)	2,036 (61%)
Nordic excl. Sweden	560 (4.2%)	138 (4.1%)
EU15 excl. Nordic	209 (1.6%)	55 (1.6%)
Europe excl. EU15 and Nordic	1,178 (8.9%)	305 (9.1%)
Asia	2,335 (18%)	600 (18%)
Africa	540 (4.1%)	135 (4.0%)
Other	317 (2.4%)	81 (2.4%)
**Education level**		
Primary	3,563 (27%)	916 (27%)
Secondary	6,065 (46%)	1,547 (46%)
Post-secondary 1	1,743 (13%)	417 (12%)
Post-secondary 2	1,809 (14%)	470 (14%)

^1^ Median (Q1, Q3); n (%)

**Fig. 1:**
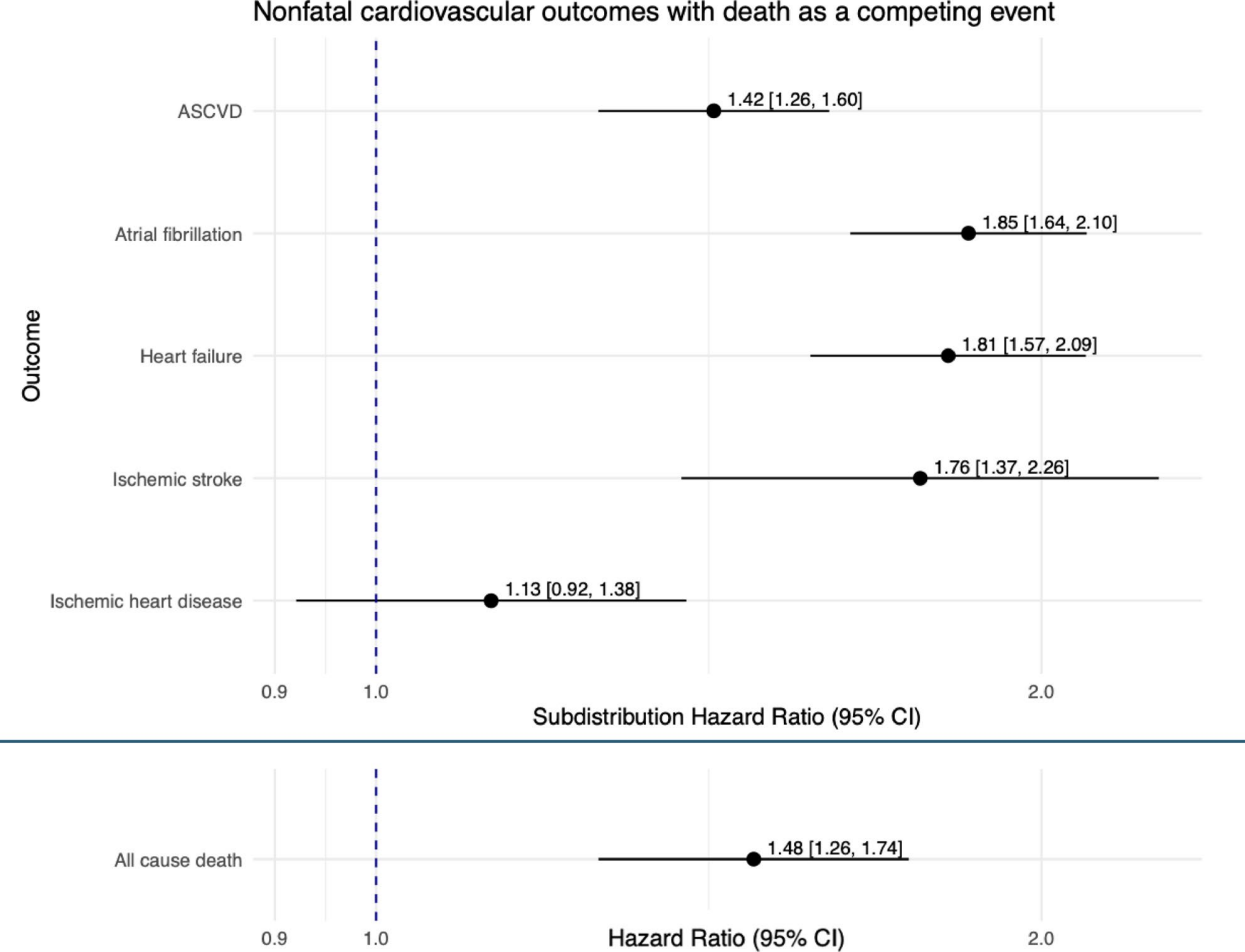
Subdistribution hazard ratios (sHR) and hazard ratios (HR) with 95% confidence intervals (CIs) for cardiovascular outcomes and all-cause death. Nonfatal cardiovascular outcomes were analysed using Fine-Gray subdistribution hazard models accounting for all-cause death as a competing event. All-cause death was analysed using a Cox proportional hazards model stratified by matched set

Our main finding was that in time-to-event analyses, severe COVID-19 was associated with an increased risk of ASCVD. Since cases also had a significantly higher risk for all-cause mortality [HR 1.48 (95% CI 1.26–1.74)], Fine-Gray subdistribution hazard models were used for non-fatal outcomes to account for death as a competing event. In the competing-risk analyses the associations remained robust and the risk of a new ASCVD event was significantly higher among cases compared to matched controls [sHR 1.42 (95% CI 1.26–1.60)]. Severe COVID-19 was further associated with increased risks of atrial fibrillation [sHR 1.85 (95% CI 1.64–2.10)] and heart failure [sHR 1.81 (95% CI 1.57–2.09)] (Fig. 1**).** Given non-proportional hazards across all outcomes, period-specific estimates for 0–1 year and 1–3 years are presented in the Supplementary [Figure S5-6] as the primary time-stratified results.

During follow-up, the incidence rate of ASCVD was 42.3 per 1,000 person-years among patients with severe COVID-19 compared with 29.0 per 1,000 person-years among controls, corresponding to an absolute rate difference of 13.3 events per 1,000 person-years.

At 3 years, the cumulative incidence of ASCVD was 10.9% among cases and 7.9% among controls (risk difference, 3% points) with most of the excess risk occurring within the first year [Table 2].

**Table 2:** Cumulative risks, risk differences, and event rates for cardiovascular outcomes and all-cause death in patients exposed to severe COVID-19 (cases, *n* = 3,350) and matched controls (*n* = 13,180)

Outcome	1 year risk (%) Cases	1 year risk (%) Controls	3-year risk (%) Cases	3-year risk (%) Controls	Risk Difference (1 vs. 3 yrs%)	Rate per 1000 patient year (*n* = 3,350 vs. 13,180)
**ASCVD**	7.1	3.6	10.9	7.9	3.4 / 3	42.3 vs. 29.0 (*n* = 364 vs. 1040)
**Atrial fibrillation**	8.5	3.4	11.1	6.3	5.1 / 4.8	43.3 vs. 22.6 (*n* = 372 vs. 826)
**Heart failure**	5.2	2.1	8.1	4.6	3.1 / 3.5	30.6 vs. 16.3 (*n* = 272 vs. 606)
**Ischemic stroke**	1.6	0.5	2.7	1.5	1.1 / 1.1	9.6 vs. 5.3 (*n* = 90 vs. 202)
**All cause death**	3.5	1.3	6.1	4.2	2.2 / 1.9	21 vs. 14.2 (*n* = 203 vs. 550)
**Ischemic heart disease**	1.9	1.3	3.6	3.2	0.6 / 0.4	12.9 vs. 11.2 (*n* = 120 vs. 421)

Risks were estimated using competing-risk cumulative incidence functions accounting for all-cause death as a competing event. For all-cause death, cumulative risk was estimated using the Kaplan-Meier method. Cumulative risks are presented as percentages at 1 and 3 years of follow-up. Risk difference represents the absolute difference in cumulative risk between cases and controls at 1 and 3 years respectively. Event rates are presented per 1,000 person-years together with the number of individuals with at least one event (first occurrence after index date and no repeated events) in cases versus controls for each outcome

**Fig. 2:**
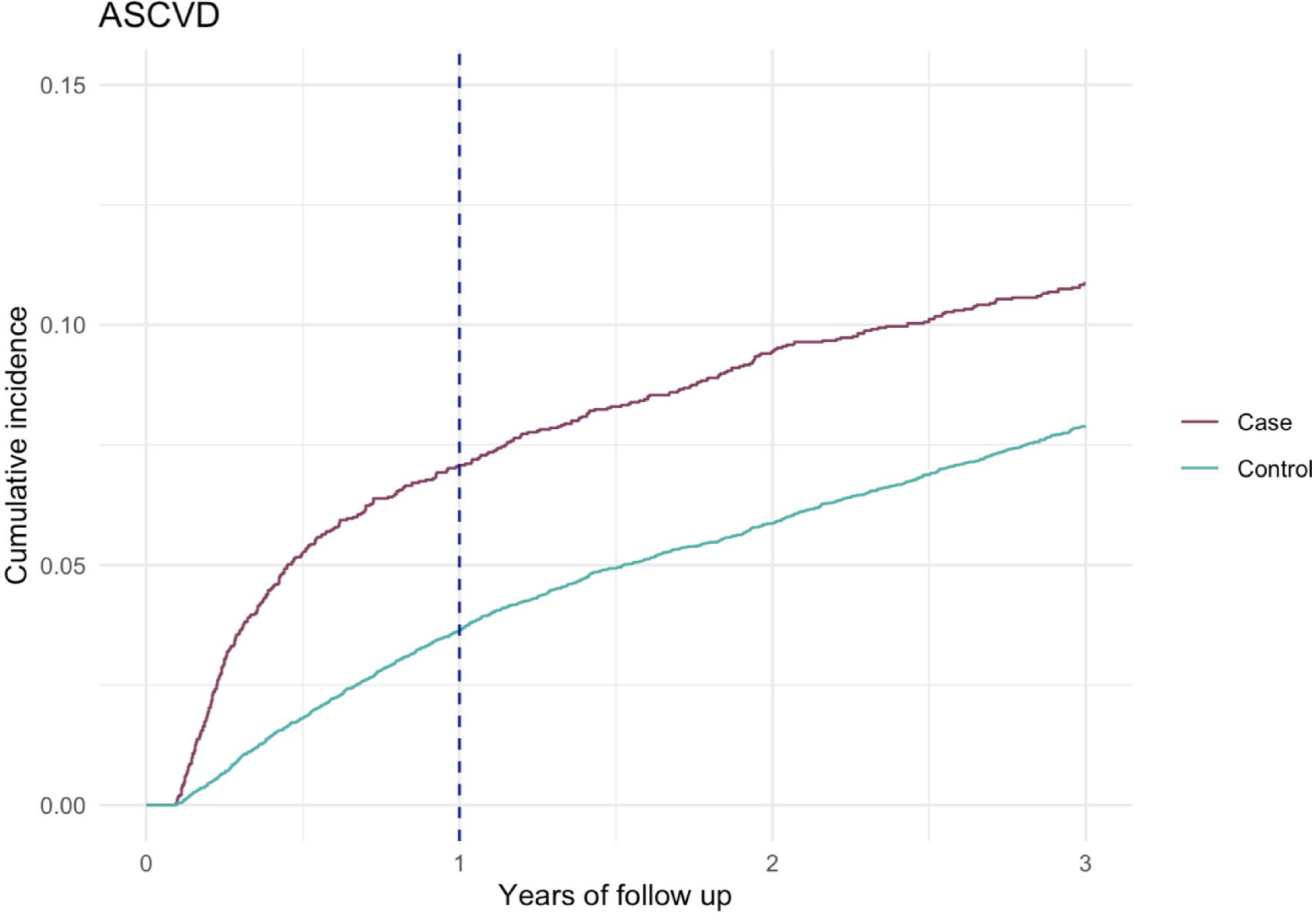
Cumulative incidence function for atherosclerotic cardiovascular disease (ASCVD) in cases and matched controls over time, accounting for all-cause death as a competing event. The vertical line indicates 1 year after ICU discharge

**Fig. 3:**
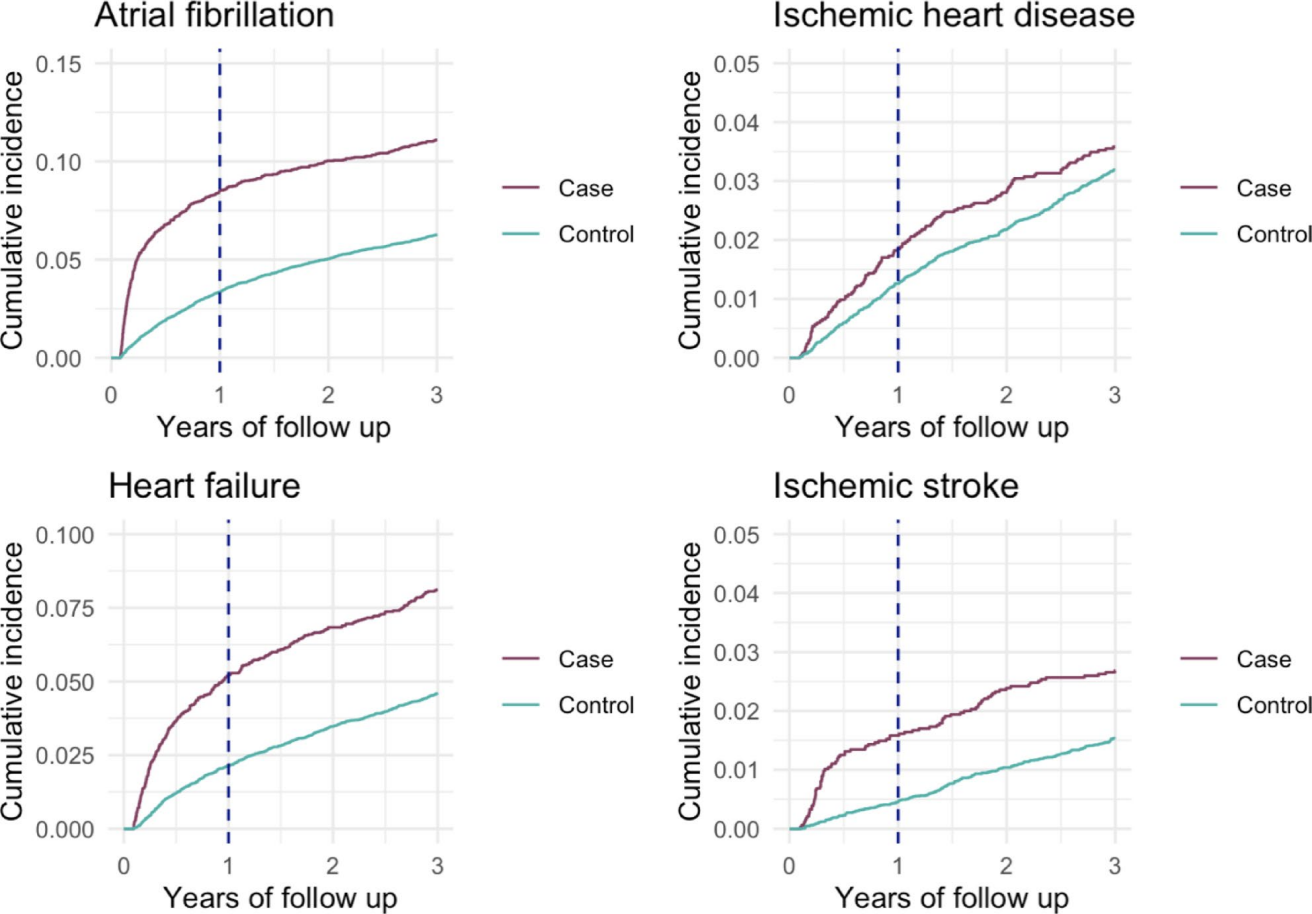
Cumulative incidence functions (CIF) for atrial fibrillation (top left), hospitalization for heart failure (bottom left), ischemic heart disease (myocardial infarction and/or angina) (top right), and ischemic stroke (bottom right), in cases and matched controls over time, accounting for all-cause death as a competing event. The vertical line indicates 1 year after ICU discharge

**Fig. 4:**
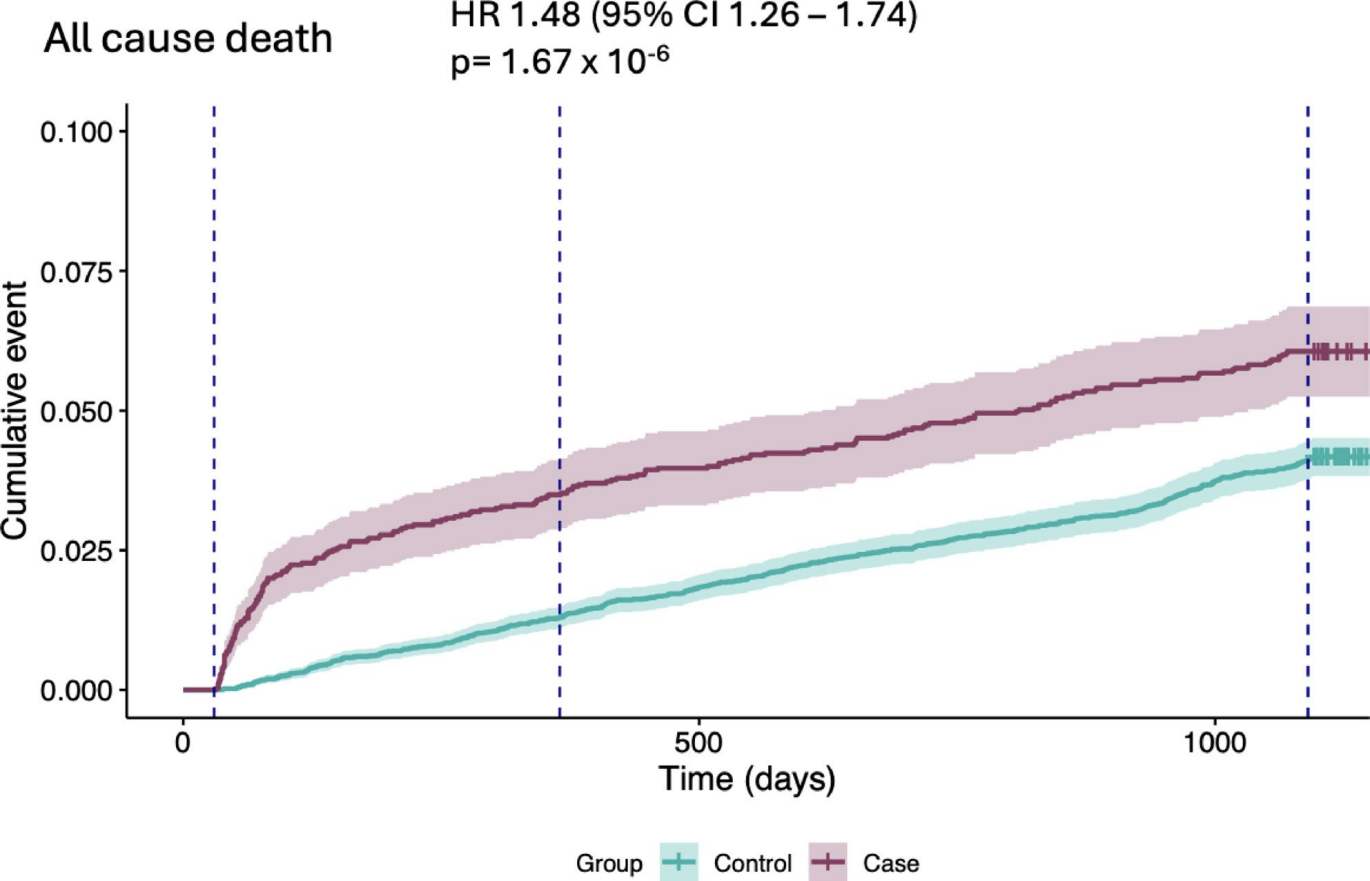
Cumulative incidence (Kaplan Meier) of all cause death in cases and matched controls over time. Shaded areas represent 95% confidence intervals. Hazard ratios (HRs) with 95% CIs and p-values are shown at 3 years of follow-up, estimated using Cox proportional hazards models stratified by matched set. Vertical lines indicate 30 days, 1 year and 3 years after ICU discharge. Tick marks indicate censoring

During the three-year follow-up, cumulative incidence plots demonstrated a persistently higher occurrence of both atherosclerotic, non-atherosclerotic cardiovascular events, and all-cause mortality among cases. The separation between curves appeared early, within the first year, and remained stable thereafter. [Figs. 2, 3 and 4]

Despite the elevated risks described above, most of both cases and controls remained free of major cardiovascular events at three years. Among those who did experience an event, atrial fibrillation and ASCVD were the dominant pathways and occurred at higher frequencies in cases [Supplementary figure S4]. Some individuals also suffered multiple CVD events during the follow-up period where atrial fibrillation and heart failure was the most common combination of diagnoses [details in Supplementary figure S7-8].

## Discussion

This nationwide matched cohort study demonstrates that individuals with severe COVID-19 had a higher observed cardiovascular risk, primarily concentrated in the early period following recovery from critical illness. In total, 4,921 ICU-treated patients with ten controls each were included, of whom 3,350 patients and 13,180 controls remained after 1:4 propensity score matching. Patients with severe COVID-19 had higher risk of ASCVD events, hospitalization with heart failure, atrial fibrillation, and all-cause mortality. This excess cardiovascular risk was mainly driven by events occurring within the first year after ICU-discharge.

To realistically estimate the longitudinal impact of COVID-19 on CVD, we included only acute CVD event and mortality occurring 30 days after ICU discharge in the analysis, as we assumed that most patients had recovered from the acute phase after this time-period. Even after this assumption, and adjustment for co-morbidities and cardiovascular risk factors through propensity score matching, cases demonstrated a higher incidence of cardiovascular events over time compared with matched controls.

Severe COVID-19 and cardiovascular disease share several major risk factors such as old age, male sex, obesity, diabetes, hypertension, and chronic kidney disease. These factors both increase the risk for severe COVID-19 and independently elevate the risk of cardiovascular events [6]. These shared risk factors may confound the association between COVID-19 severity and subsequent ASCVD and make it difficult to find the true effect of COVID-19 disease on ASCVD. We chose to propensity score match cases and controls to reduce this risk, however residual confounding due to shared risk factors cannot be fully excluded.

There is also a potential difference in surveillance where, for example, cases may have had more contact with health care after discharge and may also have been more observant on symptoms. This while controls at the same time may have avoided seeking care due to society recommendations to stay at home and fear of attracting COVID-19. However, the sustained absolute gap in cumulative incidence suggests a genuine excess burden.

When studying the cumulative incidence curves there is an early discrepancy between cases and controls for all examined cardiovascular outcomes, with most of the excess events among cases occurring within the first year. After the first year the cumulative incidence curves for cases and controls run largely in parallel, indicating similar incidence rates in the two groups thereafter. This pattern was formally confirmed by non-proportional hazards testing — Schoenfeld residuals for the Cox model and visual inspection of log-log plots for Fine-Gray models — both indicating that the excess risk is concentrated early and attenuates over time. This is consistent with an early trigger of cardiovascular events after severe COVID-19 followed by comparable slopes of the curves beyond the first year. Several mechanisms could account for this excess risk. First, COVID-19 may trigger subclinical pre-existing atherosclerotic disease or second, accelerate atherothrombotic processes through systemic inflammation and endothelial injury [22–24]. Also, myocardial inflammation may promote arrhythmogenic changes and autonomic dysfunction [5, 25] while ICU specific exposures (immobility, fluid shifts, prolonged catecholamine exposure, vasopressors, and mechanical ventilation) may amplify these effects [26]. The observed associations may partly reflect the effects of critical illness and intensive care management, including mechanical ventilation, rather than COVID-19 effects alone. As such, unravelling the impact of COVID-19 itself, remains challenging.

Despite the exclusions made in our study design (excluding all patients that died within 30 days after discharge) all-cause mortality remained higher in cases. This suggests that the increased mortality is not solely driven by acute complications to the severe infection but may instead be due to long-term consequences of COVID-19. However, all-cause mortality is a complex and relatively unspecific endpoint, influenced not only by cardiovascular disease that this study aimed to study. Therefore, this parameter should be interpreted with caution. Still, the finding highlights the long-term vulnerability among patients that suffered severe COVID-19 is of clinical relevance and offers further investigation.

## Conclusion

Our findings suggest that patients who survived severe COVID-19 are a vulnerable group with excess risk for major cardiovascular events and mortality even after the acute phase but mainly during the first year after discharge. This highlights the long-term systemic effects of COVID-19, potentially mediated by persistent inflammation and endothelial dysfunction, and this high-risk group should be subject for targeted cardiovascular surveillance and secondary prevention.

## Limitations

Although we have used mandatory, high-quality nationwide registries, the study has several limitations. First, controls were not treated in the ICU for COVID-19, but we lack data regarding non-ICU COVID-19, especially milder infections that were not captured in our registries due to limited testing resources in the early pandemic. Hence, misclassification of exposure among controls is possible, as some individuals may have had a prior COVID-19 infection. This may have reduced the observed difference between groups and more likely underestimated the true difference rather than overestimated. If controls had mild COVID-19 they may also have elevated cardiovascular risk, decreasing the observed difference.

Second, we lack data from primary health care facilities, and this may cause an underestimation of cardiovascular endpoints in both cases and controls. However, it is unlikely that our targeted diagnoses are set in primary care facilities. A further limitation is that data on hospital discharge dates were unavailable; consequently, we could not determine the interval between ICU discharge and hospital discharge for all cases. It is therefore possible that a proportion of early post-ICU, in-hospital events were captured within the follow-up period. This should be considered when interpreting outcome rates in the immediate period following the index date. Finally, although matching reduce confounding by demographics, co-morbidities, and socioeconomic variables it is still possible that both residual confounding and surveillance bias occur. Finally, as an observational study, causal inference cannot be established.

## Supplementary Material

Supplementary Material 1.

## Data Availability

The datasets used and analysed during the current study are not publicly available due to legal and ethical restrictions. Access to the data requires approval from the relevant register holders and an appropriate ethical permit.

## Abbreviations

AHA: American Heart Association
ASCVD: Atherosclerotic cardiovascular disease
AF: Atrial fibrillation
CI: Confidence interval
CIF: Cumulative incidence function
COPD: Chronic obstructive pulmonary disease
COVID-19: Coronavirus disease 2019
CVD: Cardiovascular disease
ESC: European Society of Cardiology
HR: Hazard ratio
ICU: Intensive care unit
IHD: Ischemic heart disease
KM: Kaplan-Meier
SIR: Swedish Intensive Care Registry
sHR: Subdistribution Hazard ratio
WHO: World Health Organization
CKD: Chronic kidney disease
HF: Heart failure
ICD: International Classification of Diseases
IQR: Interquartile range
