# Supplementary material for: Beyond recovery: long-term cardiovascular risks after severe COVID-19 requiring intensive care
Source: Crit Care. 2026 May 25;30:272. doi: 10.1186/s13054-026-06088-5 (PMC13202947; doi:10.1186/s13054-026-06088-5)
Supplement: Supplementary file 1 — Supplementary Material 1. [file 13054_2026_6088_MOESM1_ESM.docx]

# Supplementary

[Supplementary 1](#_Toc228705247)

[Flow chart for inclusion and exclusion. 2](#_Toc228705248)

*Codes (ICD-10) used for diagnoses ……………………………………………………………………………………2*

[Covariate Balance of the 1:4 matched cases and controls. 3](#_Toc228705249)

[Registries and Data collection 4](#_Toc228705250)

[Definition of covariates and variables for analyses 4](#_Toc228705251)

[Clinical outcomes observed during the three-year follow up 5](#_Toc228705252)

[Cause-specific analyses — Kaplan-Meier failure curves (death censored) with cause-specific hazard ratios for non-fatal cardiovascular outcomes. 5](#_Toc228705253)

[Distribution of CVD with multiple outcomes possible 7](#_Toc228705254)

[References 8](#_Toc228705255)

### Flow chart for inclusion and exclusion.


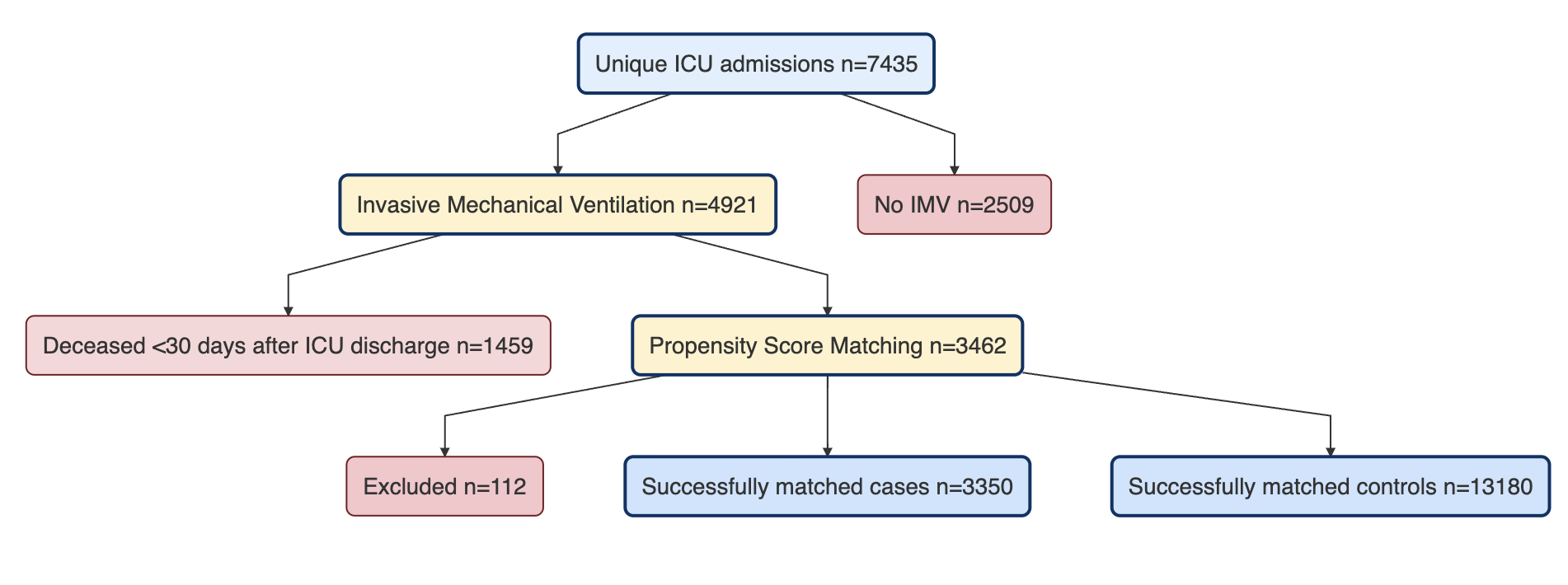


Supplementary Figure S1.

Codes (ICD-10) used for diagnoses.

| **DIAGNOSE** | **ICD-code** |
| --- | --- |
| Diabetes Mellitus 1 | E10 |
| Diabetes Mellitus 2 | E11 |
| Obesity | E66 |
| Lung embolism | I26 |
| Venous thromboembolism | I80\|I82 |
| Chronic kidney failure | N18 |
| Heart failure | I50\|I51\|I52 |
| Atrial fibrillation/flutter | I48 |
| Arrest | I46 |
| Peri-myocarditis | I30\|I31\|I32\|I40 |
| COPD | J44 |
| Asthma | J45 |
| Rheumatoid arthritis | M05\|M06 |
| Systemic inflammatory disease | M30\|M31\|M32\|M33\|M34\|M35\|M36 |
| Ischemic heart disease | I20\|I21\|I22\|I23\|I24\|I25 |
| Hypertension | I10\|I11 |
| Covid-19 | U071 |
| Intracranial haemorrhage | I60\|I61\|I62 |
| Ischemic stroke | I63\|I64\|I65\|I66\|I67 |

Figure S2.

ASCVD was defined in accordance with the European Society of Cardiology (ESC) concept of clinical atherosclerotic cardiovascular disease (1) and included ischemic heart disease and ischemic cerebrovascular disease. Ischemic heart disease was identified using ICD codes I20-I25, and ischemic stroke using I63-67. Haemorrhagic stroke and non-atherosclerotic cardiovascular conditions, such as heart failure (I50) and atrial fibrillation (I48) were not included but identified separately.

### Covariate Balance of the 1:4 matched cases and controls.


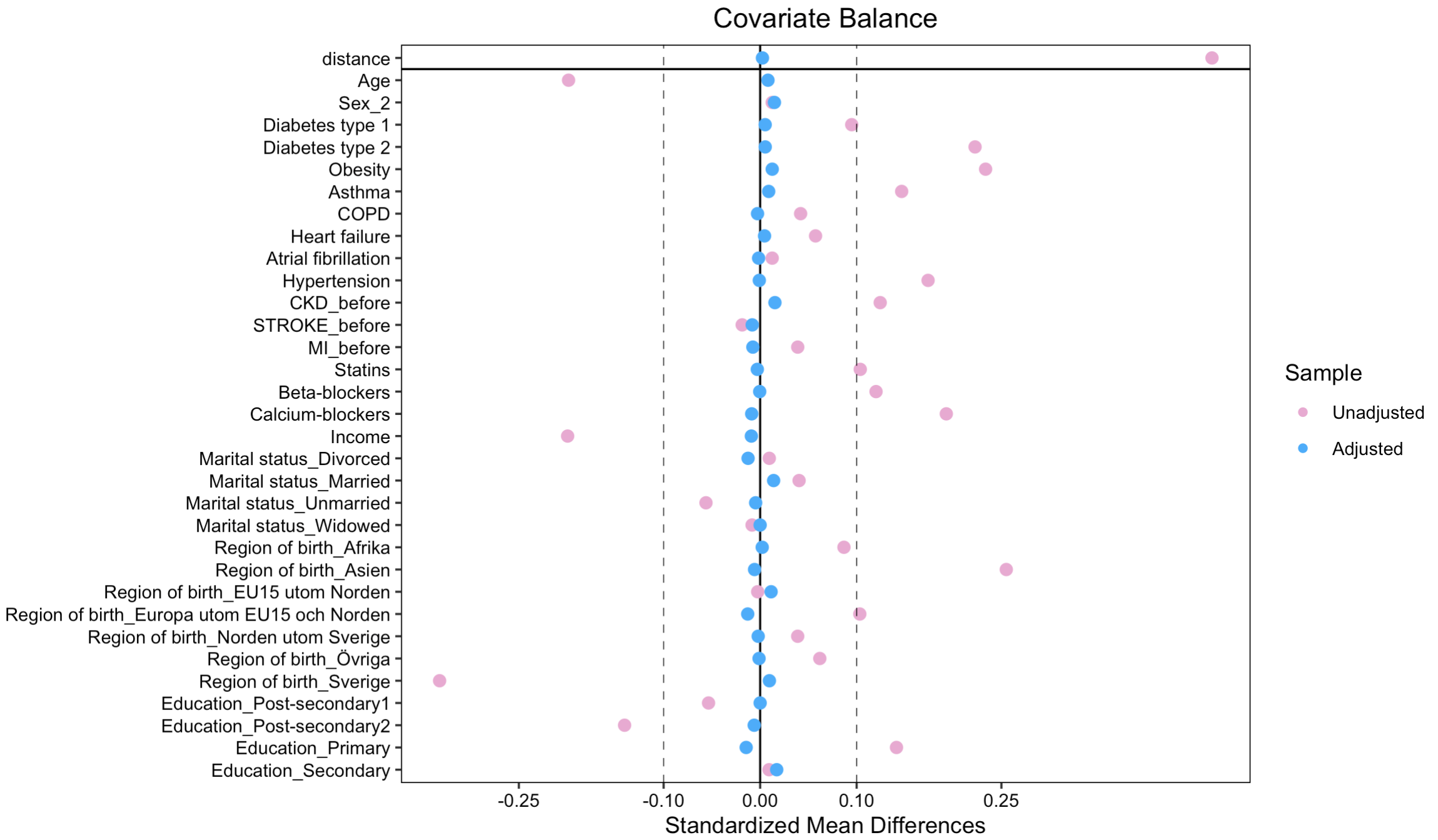


Supplementary Figure S3.

### Registries and Data collection

We used multiple Swedish national registries using everyone’s unique personal identification number. As primary data source and for patient identification we used SIR (2). With a >95% coverage of nationwide ICU admissions we received date of admission, mode of ventilation and ICU outcomes. The Swedish Longitudinal Integrated Database for Health Insurance and Labour Market Studies (3) provided data on socioeconomic variables and sociodemographic variables, including region of birth, level of education and income status.) The National Patient Registry (4) provided data on inpatient care in Sweden and through this registry we collected all medical diagnoses according to the International Classification of Diseases, version 10 (ICD-10) for both cases and control subjects. The Swedish Prescribed Drugs Register (5) provided all dispensed prescribed drugs for our cases and controls according to the Anatomical Therapeutic Chemical Classification (ATC-code). The Swedish Cause of Death Registry (6) was used for determining date of death and cause of death.

### Definition of covariates and variables for analyses

Level of education, based on the highest completed level was categorized as: primary education (≤ 9 years), secondary education (10-12 years) and post-secondary education (>12 years). Region of birth was defined as born in 1) Sweden, 2) a Nordic country (excluding Sweden), 3) European Union 15, 4) another EU country, 5) Asia, 6) Africa, 7) South America, or 8) other region of birth (North America, Oceania, and former Soviet Union). Region of birth, sex (male/female), and age constituted variables for subgroup analyses. Based on the household’s disposable income we categorized everyone into three groups.

### Clinical outcomes observed during the three-year follow up


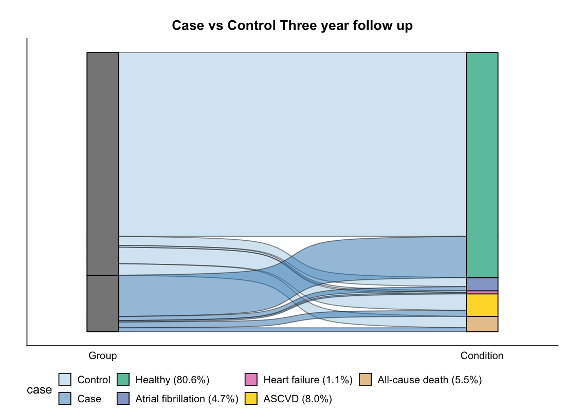


Supplementary Figure S4. Sankey plot illustrating the distribution of outcomes at three years of follow-up among cases and controls. The right-hand axis represents the first recorded outcome during follow-up. Percentages reflect the proportion of cases experiencing each outcome as their first event and should be interpreted accordingly, as patients may have experienced multiple events during follow-up. Healthy individuals had no recorded outcome event during the follow-up period, and this percentage is accurate although it includes both cases and controls.

### Cause-specific analyses — Kaplan-Meier failure curves (death censored) with cause-specific hazard ratios for non-fatal cardiovascular outcomes.

As a complementary cause-specific analysis, Kaplan–Meier failure curves (death censored) are presented below. In contrast to the Fine–Gray subdistribution hazard models used in the main manuscript, which retain individuals who die in the risk set and estimate absolute risk in the presence of competing events, these cause-specific analyses treat death as a censoring event. They therefore reflect the hazard of each outcome among survivors and should be interpreted alongside, not instead of, the competing-risk estimates.


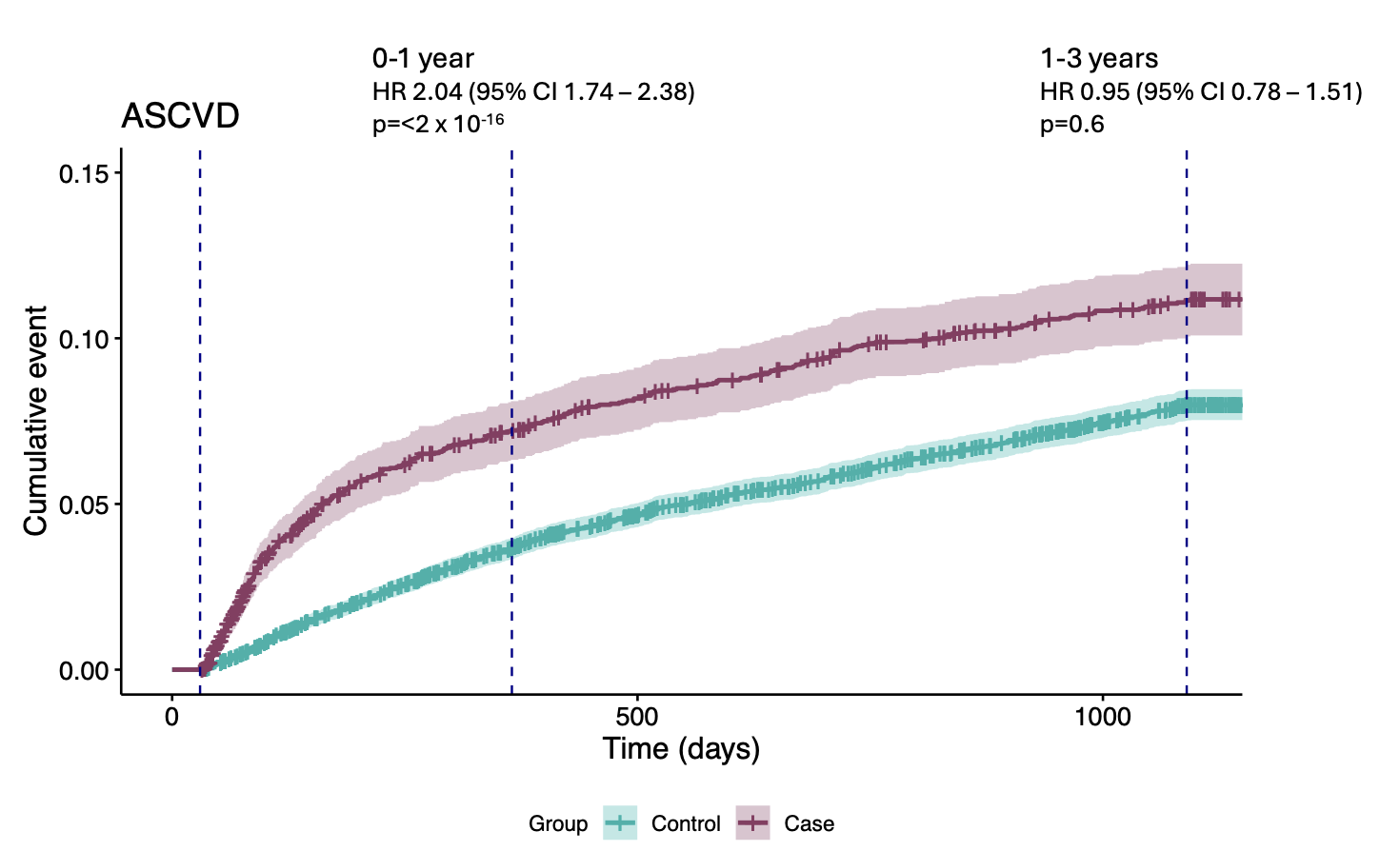


Supplementary Figure S5. Kaplan-Meier cumulative incidence curves for atherosclerotic cardiovascular disease (ASCVD) in cases and matched controls. Cause-specific hazard ratios (HR) with 95% confidence intervals and p-values are shown for 0–1 year and 1–3 years of follow-up, estimated using Cox proportional hazards models stratified by matched set. Death was treated as a censoring event. Dashed vertical lines indicate 30 days, 1 year and 3 years after ICU discharge. Tick marks indicate censoring.


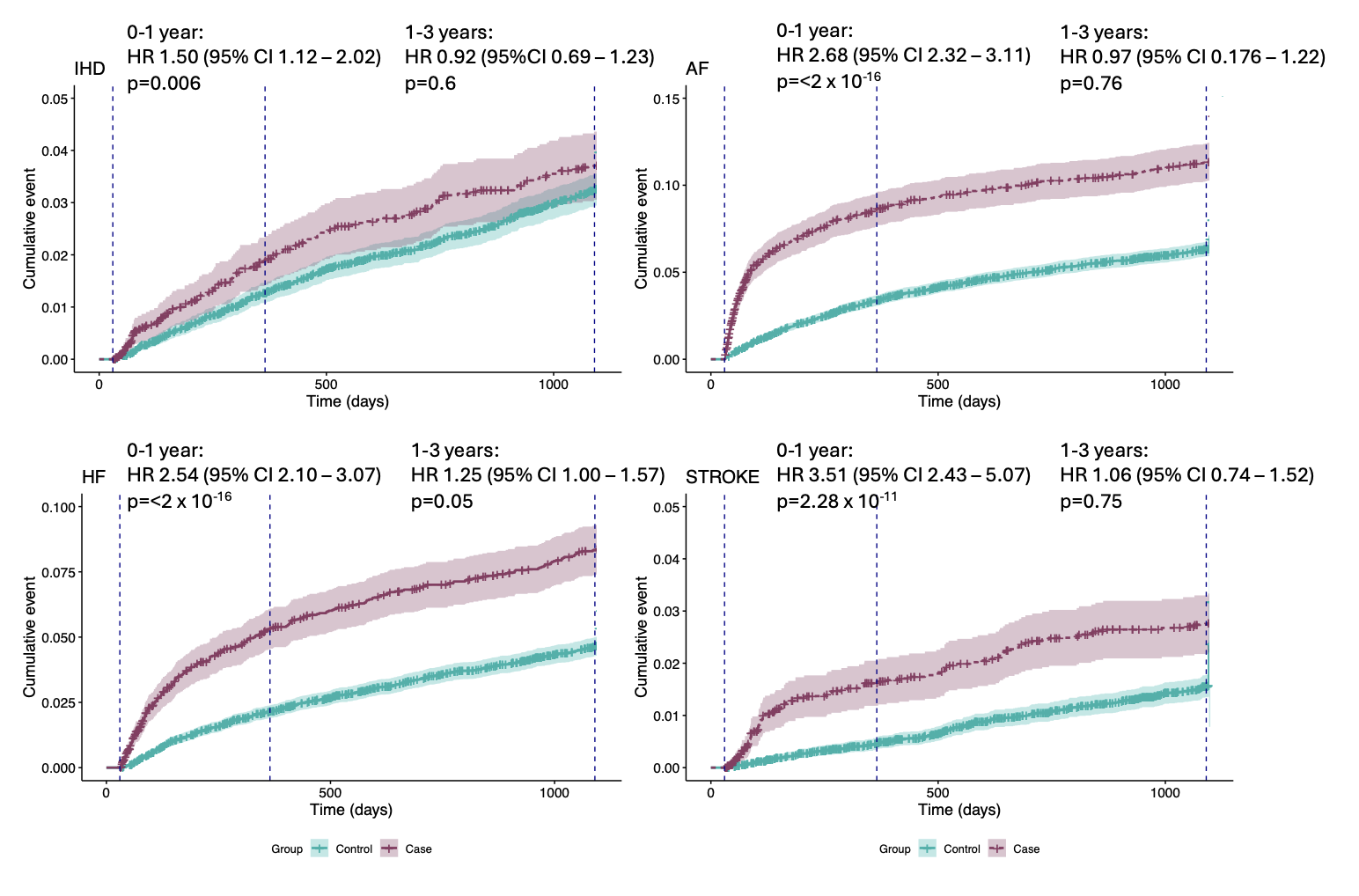


Supplementary Figure S6. Kaplan-Meier cumulative incidence curves for ischemic heart disease (top left), atrial fibrillation (top right), heart failure (bottom left) and ischemic stroke (bottom right) in cases and matched controls. Cause-specific hazard ratios (HR) with 95% confidence intervals and p-values are shown for 0–1 year and 1–3 years of follow-up, estimated using Cox proportional hazards models stratified by matched set. Death was treated as a censoring event. Dashed vertical lines indicate 30 days, 1 year and 3 years after ICU discharge. Tick marks indicate censoring.

### Distribution of CVD with multiple outcomes possible


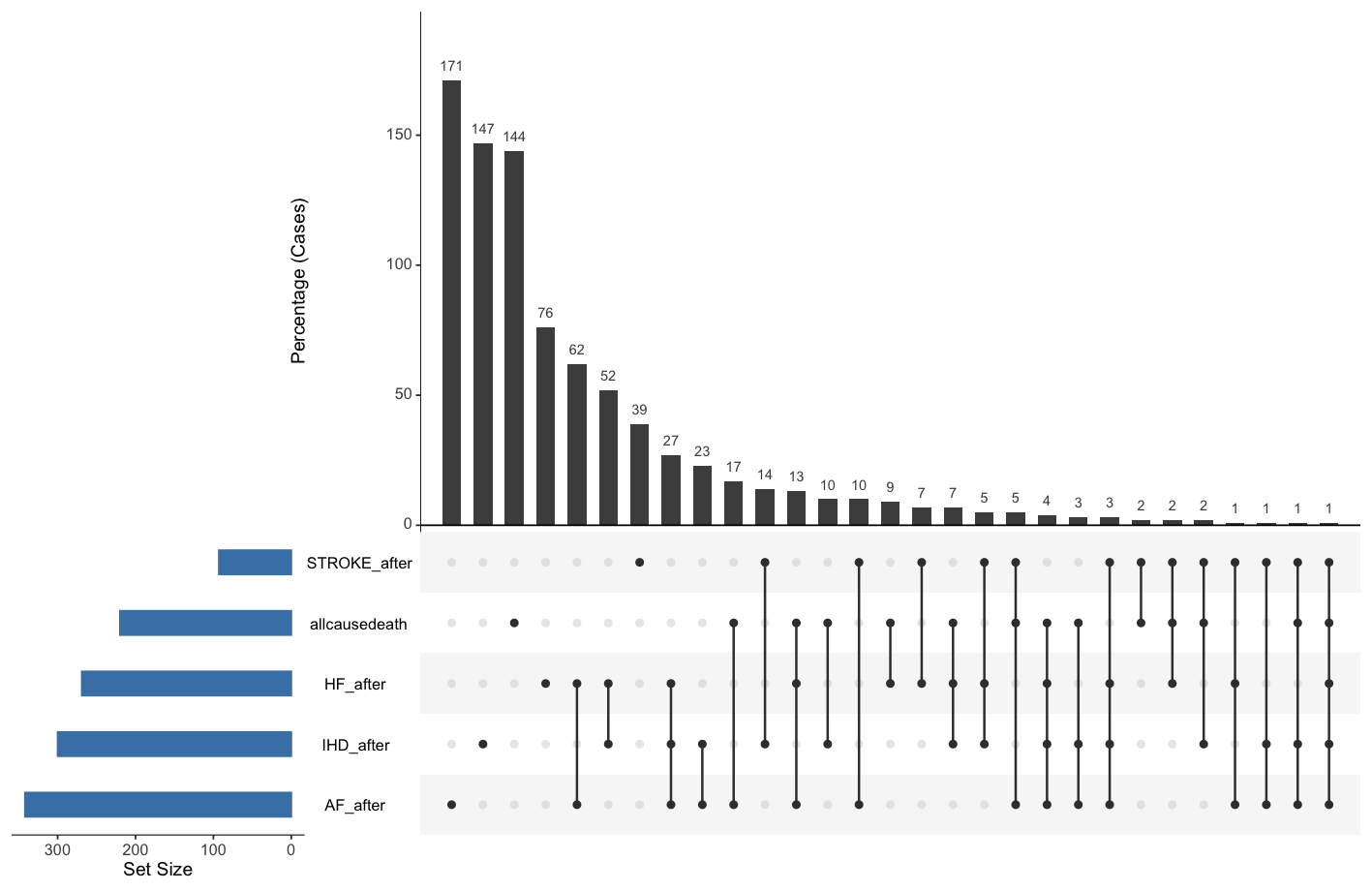


Supplementary figure S7. Cases


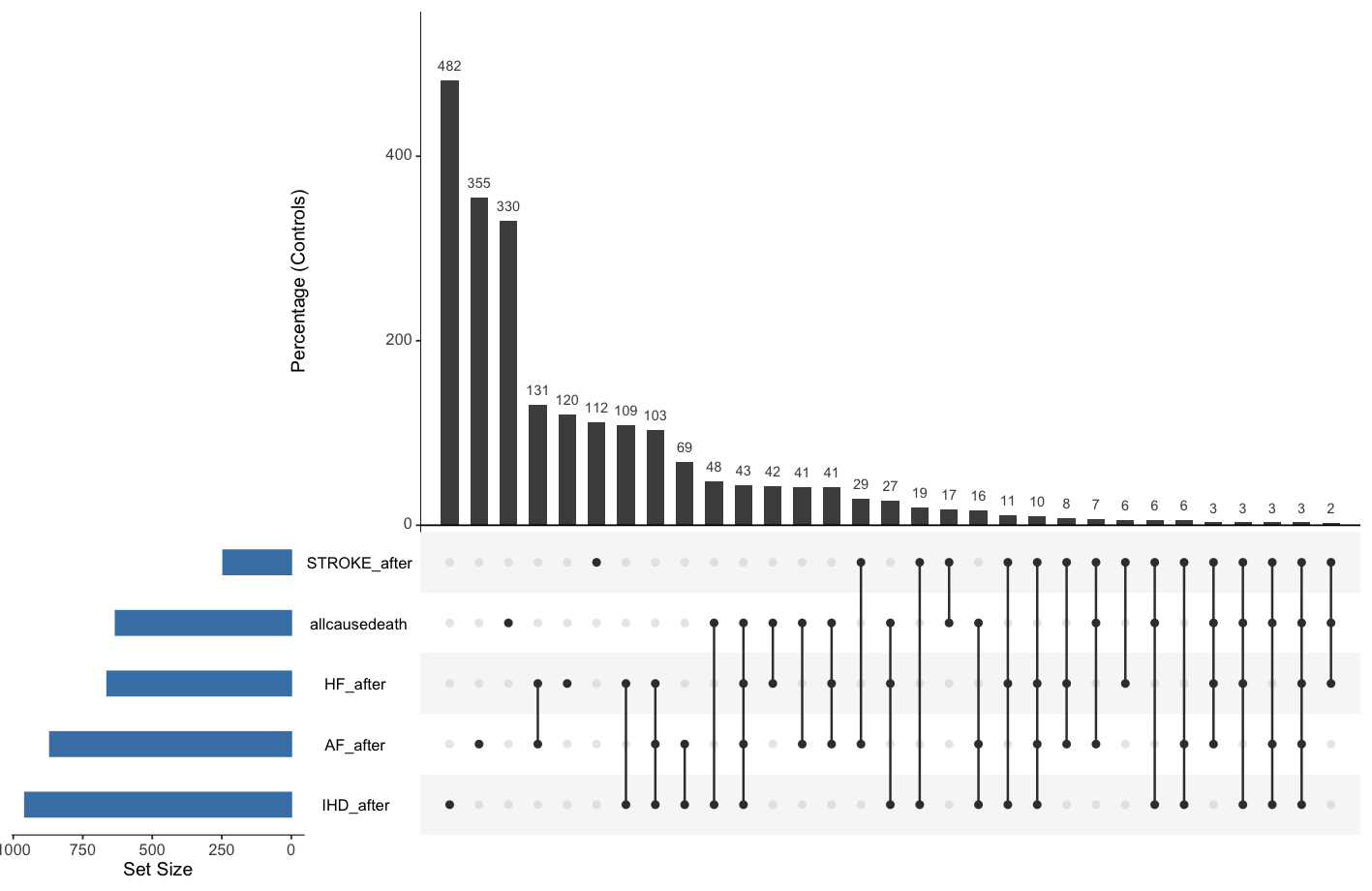


Supplementary figure s8. Controls

### References

1. Visseren FLJ, Mach F, Smulders YM, Carballo D, Koskinas KC, Bäck M, et al. 2021 ESC Guidelines on cardiovascular disease prevention in clinical practice. Eur Heart J. 2021;42(34):3227-337.

2. Care SrfI. SIR Stockholm: SKR; 2021 [Available from: <https://www.icuregswe.org>.

3. SCB. Internet SCB: SCB; 2025 [Available from: <https://www.scb.se/vara-tjanster/bestall-data-och-statistik/register/lisa/>.

4. Ludvigsson JF, Andersson E, Ekbom A, Feychting M, Kim J-L, Reuterwall C, et al. External review and validation of the Swedish national inpatient register. BMC public health. 2011;11:450.

5. Socialstyrelsen. National Prescribed Drug Register [Available from: <https://www.socialstyrelsen.se/en/statistics-and-data/registers/national-prescribed-drug-register/>.

6. Socialstyrelsen. National Cause of Death Register [Available from: <https://www.socialstyrelsen.se/en/statistics-and-data/registers/national-cause-of-death--register/>.
